# [Expression of Concern] Inhibitory effect of curcumin on angiogenesis in ectopic endometrium of rats with experimental endometriosis

**DOI:** 10.3892/ijmm.2026.5787

**Published:** 2026-03-09

**Authors:** Ying Zhang, Hong Cao, Yue-Yue Hu, Hua Wang, Chang-Jun Zhang

Int J Mol Med 27: 87-94, 2011; DOI: 10.3892/ijmm.2010.552

Following the publication of the above paper, it was drawn to the Editor's attention by a concerned reader that, regarding the histopathological images shown in Fig. 3 on p. 90, Fig. 3E and F showed an overlapping section, suggesting that these data panels were derived from the same original source where different treatment groups were reported.

The authors have been contacted by the Editorial Office to offer an explanation for this apparent anomaly in the presentation of the data in this paper, and we are awaiting their response. Due to the fact that we have been made aware of potential issues surrounding the scientific integrity of this paper, we are issuing an Expression of Concern to notify readers of these potential problems while the Editorial Office continues to investigate this matter further.

